# Effects of electrical-hydrothermal aging degradation on dielectric and trap properties of high temperature vulcanized silicone rubber materials

**DOI:** 10.1039/d0ra00134a

**Published:** 2020-01-22

**Authors:** Huasong Xu, Congzhen Xie, Rui Wang, Bin Gou, Shoukang Luo, Jiangang Zhou

**Affiliations:** School of Electric Power, South China University of Technology Guangzhou 510641 China congzhen168@163.com

## Abstract

In this paper, a new aging platform combined the high voltage electric field and the hydrothermal environment was built. To investigate the aging mechanism, physicochemical, dielectric and trap properties of HTV SR before and after electrical-hydrothermal aging for 24 days were discussed. The results indicated that, compared with hydrothermal aging, more cracks and holes appeared on the surface of HTV SR after electrical-hydrothermal aging, and the content of flame retardant decreased significantly. Due to the main chain and side chain scission of PDMS, lots of low weight molecular (LWM) substances and free radicals were produced. And the tensile strength and elongation at break significantly decreased. Various physical and chemical defects appeared in the HTV SR specimen in the process of electrical-hydrothermal degradation, as a result of which, the dielectric constant significantly increased and the peak trap density increased by about 2.5 times compared to the virgin sample. The increase in trap density in turn accelerated the charge accumulation and enhanced the breakdown probability, resulting in the electric field strength decrease from 21.8 kV mm^−1^ to 16.1 kV mm^−1^ and severe degradation of HTV SR.

## Abstract 2

In this paper, a new aging platform combined the high voltage electric field and the hydrothermal environment was built.

## Graphical abstract

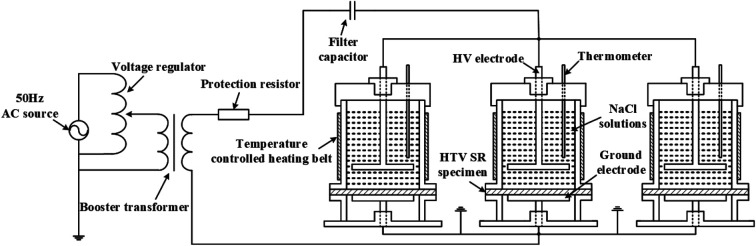

## 1 Introduction

Composite insulators are widely used in power systems because of their light weight and strong resistance to pollution flashover.^1–8^ As an important material for the sheaths and sheds of composite insulators, high temperature vulcanized silicone rubber (HTV SR) plays an important role. However, as the operating life increases, the aging problem becomes more and more serious. Due to long-term exposure to the harsh environment, external insulation of composite insulators can suffer from multiple stresses such as electrical discharge, temperature and mechanical stress *et al.*^9–13^ In hot and humid areas all over the world, they are also accompanied by the problem of hydrothermal aging. Therefore, it is of great significance to carry out multiple stress aging research on external insulation materials of composite insulators for mastering the aging rule and evaluating the insulation state.

Degradation studies of HTV SR have been a topic for research from the last few decades. Z. D. Jia *et al.*^14^ studied the thermo-oxidative aging process of HTV SR and calculated the activation energy of the aged specimens. Z. Wang *et al.*^15^ investigated the absorption characteristics of HTV SR in deionized water, NaCl solution and HNO_3_ solution, analyzing the effects of the three liquids on the electrical properties. Mansab Ali and Reuben Hackam^16^ studied the loss of hydrophobicity of HTV SR after immersion in saline solutions at different temperatures, revealing that the contact angle will decrease sharply during immersion. Yong Zhu *et al.*^17^ described the degradation of HTV SR resulting from corona discharges under atmospheric pressure, showing that the corona discharges can cause physical and chemical damages to the surface of HTV SR. Recent studies focus on corona aging, thermal aging, electrical branch aging and other single physical field aging mechanism. Comparative analysis of hydrothermal and electrical-hydrothermal aging has not been reported. However, as sheds of composite insulators, HTV SR has been exposed to multiple stresses, and the aging rate is much higher than the single field aging. Therefore, studying the electrical-hydrothermal aging properties to analyze the aging mechanism is imperative.

In the present work, the aging mechanism of HTV SR exposed to multiple stresses has not been clearly claimed. In this paper, AC breakdown field strength, dielectric spectroscopy, thermally stimulated current (TSC), Fourier transform infrared (FTIR) spectroscopy, scanning electron microscopy (SEM) and energy dispersive X-ray (EDAX) analysis were conducted to analyze changes of electrical characteristics and physical properties after hydrothermal aging and electrical-hydrothermal aging. Subsequently, the mechanism of electrical-hydrothermal aging was analyzed.

## 2 Experimental details

### 2.1 Sample preparation

The HTV SR specimen was provided by Dongguan Gaoneng Company in China. It should be emphasized that the composite insulator manufactured by the Dongguan Gaoneng Company has been running successfully in hot and high humidity areas in southern China for nearly 20 years. The formulation of the HTV SR specimen was 100 parts per hundred (phr) methyl vinyl silicone rubber, 40 phr meteorological silica, 125 phr aluminum powder, 6 phr silicone oil, 2 phr vulcanizing agent and some iron oxide colorant. The methyl vinyl silicone rubber, meteorological silica, aluminum powder, silicone oil and colorant are added to an open mill in proportion and mixed uniformly, then heated in an oven at 150 °C for 2 hours and added vulcanizing agent at room temperature. After the vulcanizing agent is mixed in the open mill, the compound is vulcanized and formed on a flat vulcanized machine. Finally, the HTV SR with a diameter of 100 mm and a thickness of 2 mm is prepared. The detailed product information (code, supplier) of each component for the formulation of the HTV SR is listed in Table 1. Outside of China, the similar HTV SR specimen can be easily procured in factories that produce composite insulators. For instance, Mar-Bal company in America, Maschinenfabrik Reinhausen company in German, *etc.*

**Table 1:** Production information of each component for the formulation of the HTV SR specimen

Component	Supplier
Methylvinyl silicone rubber	Jinjinle Chemical Co., Ltd. China
Meteorological silica	Degussa AG, America
Aluminum powder	Yuanyang Powder Technology Co., Ltd. China
Silicone oil, vulcanizing agent, iron oxide colorant	Gaide Chemical Co., Ltd. China

### 2.2 Test apparatus and test method

**Fig. 1:**
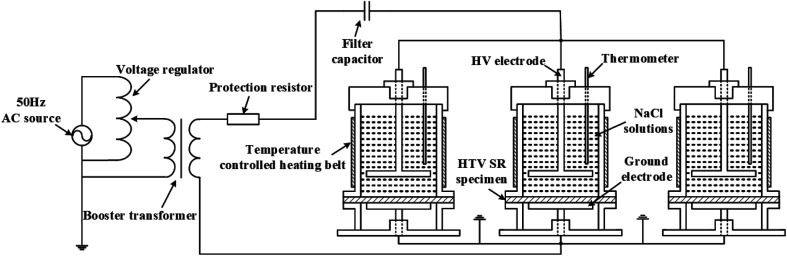
The experimental set up for the electrical-hydrothermal aging effect.

**Fig. 2:**
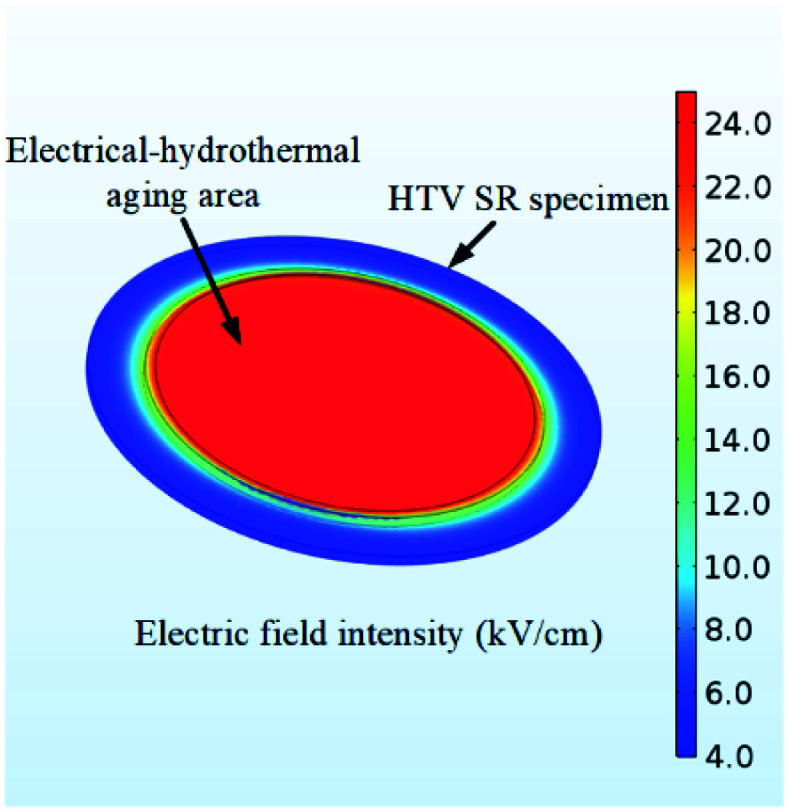
Characteristics of the surface electric field intensity at 25 kV cm^−1^ on the HTV SR specimen.

The experimental set up for investigating the electrical-hydrothermal aging effect is shown in Fig. 1. It is composed of AC power box, rod-plate electrode, quartz tube and heating plate. Saline solutions were prepared as the conductive solution and the salt contaminant. The high voltage electrode, when immersed in the saline solutions, will provide a uniform and stable electric field on the HTV SR. To verify the electric field uniformity, the simulation model was established using the COMSOL and the results were shown in Fig. 2. As shown in Fig. 2, the HTV SR specimens were in a uniform electric field. In the electrical-hydrothermal aging test, the temperature of the heating plate was controlled at 30 °C, 60 °C, 80 °C and the electric field intensity was 0 kV cm^−1^ (equivalent to hydrothermal aging), 15 kV cm^−1^ and 25 kV cm^−1^. For comparison, the temperature of the saline solutions in the hydrothermal aging test was also controlled at 30 °C, 60 °C and 80 °C.

### 2.3 Characterization

#### 2.3.1 Rate of weight change

The initial weight of the HTV SR was measured using an electronic precision balance with a measurement accuracy of 0.1 mg. After aging for several days, the HTV SR with a diameter of 100 mm and a thickness of 2 mm was taken out and cleaned with anhydrous ethanol. Then, the weights of the HTV SR were measured at room temperature and humidity. When the indication of the electronic precision balance is stable, the weights of each HTV SR specimen were recorded. The rate of weight change can be calculated as the following equation:1
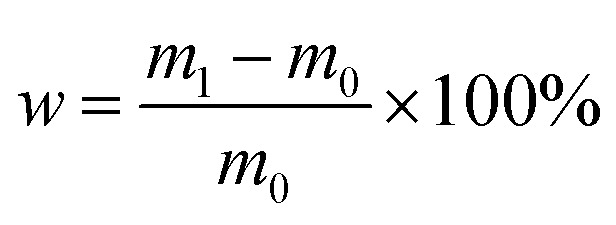
where *m*_1_ and *m*_0_ are the weight at aging time *t* and the initial weight.

#### 2.3.2 Measurements of electrical characteristics

To analyze the electrical characteristics under different aging behavior, the AC breakdown voltage, the complex dielectric constant and the thermally stimulated current were measured before aging and at different states of the aging process.

**Fig. 3:**
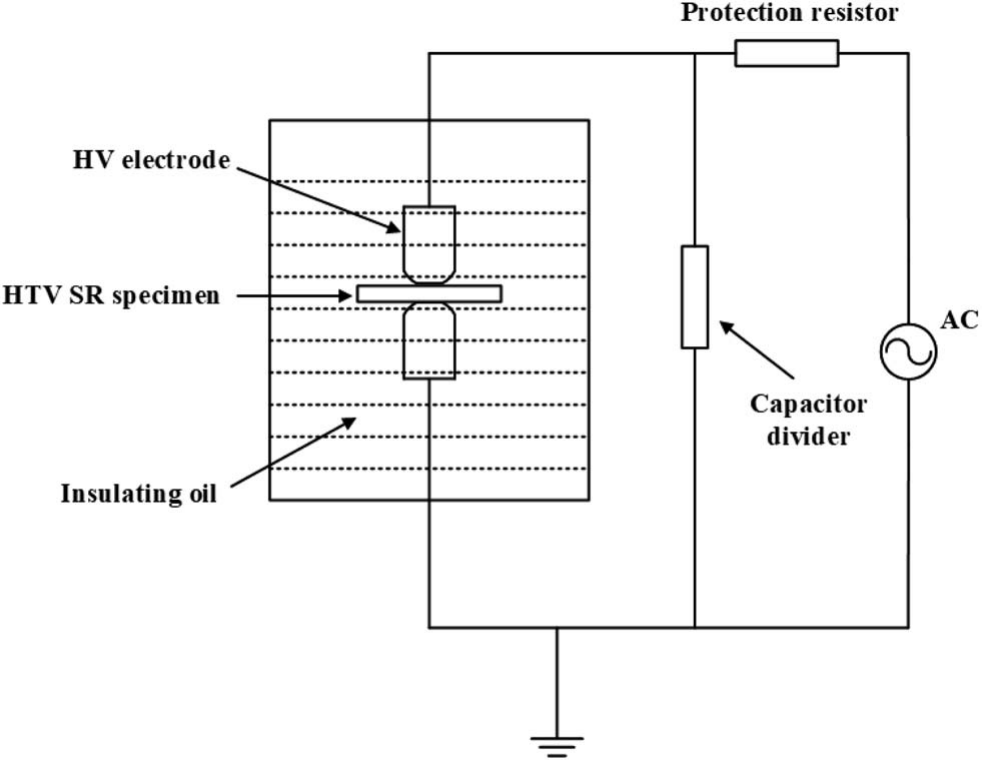
Experimental setup for the AC breakdown studies.

The AC breakdown voltage measurements were performed on the hydrothermal and electrical-hydrothermal aging specimens. In the measurement of AC breakdown voltage, a set of 5 samples with a diameter of 100 mm and a thickness of 2 mm were tested to calculate the breakdown field strength. The experimental setup for the AC breakdown voltage was shown in Fig. 3. The aging specimens are placed in the plate to plate arrangement, and placed into a vessel containing filling with insulation oil to avoid surface discharges. During the test, the voltage is increasing step by step until the breakdown occurs. The voltage was rising at the rate of 500 V s^−1^. When the breakdown occurs, the AC breakdown stress can be calculated according to the equation *E* = *V*/*d*, where *V* is the breakdown voltage and *d* is the thickness of the aging specimen in the breakdown point.

For the investigation of the frequency domain dielectric spectroscopy, the dielectric constant and loss of dielectric materials at different frequencies were analyzed. In this paper, the dielectric spectroscopy of different aging specimens with a diameter of 40 mm and a thickness of 2 mm was measured by ALPHA-ANB type broadband dielectric spectrometer produced by Guangzhou Huayu Electronic Service Co., Ltd. Each HTV SR specimen in the study, the dielectric response in the range of frequency at 10^0^ Hz to 10^5^ Hz was measured at room temperature.

For the measurement of TSC, the HTV SR specimen with a thickness of 2 mm and a diameter of 40 mm were vacuum vaporized with an Au electrode on both sides. Firstly, the HTV SR specimen was loaded between the electrodes and heated to 50 °C. Secondly, the DC voltage of 3 kV was applied to the HTV SR and kept warm for 20 min. Then the HTV to −50 °C quickly with liquid nitrogen. After keeping the temperature at −50 °C and the voltage at 3 kV for 2 min, the polarization voltage can be removed. When the current decays to zero, the specimen can be heated at a rate of 5 °C min^−1^. In the process, the depolarization current was measured as a function of temperature.

#### 2.3.3 Measurements of physicochemical characteristics and mechanical characteristics

In the measurements of physicochemical properties, FTIR spectroscopy (VERTEX 70) was applied to study the surface chemical groups of initial samples, hydrothermal aging and electrical-hydrothermal aging samples. To study the surface aging degree, SEM (LEO 1530VP), outfitted with the EDAX detector to recognize the elements, was employed to compare the effects in different aging mode. The above testing samples are all 5 mm in diameter and 2 mm in thickness. Tensile properties were measured using the Electro-Mechanical Universal Testing Machines. The stretching rate was about 50 mm min^−1^. Each HTV SR sample is 20 mm in length, 10 mm width and 2 mm in thickness.

## 3 Results and discussion

### 3.1 Relative weight change

**Fig. 4:**
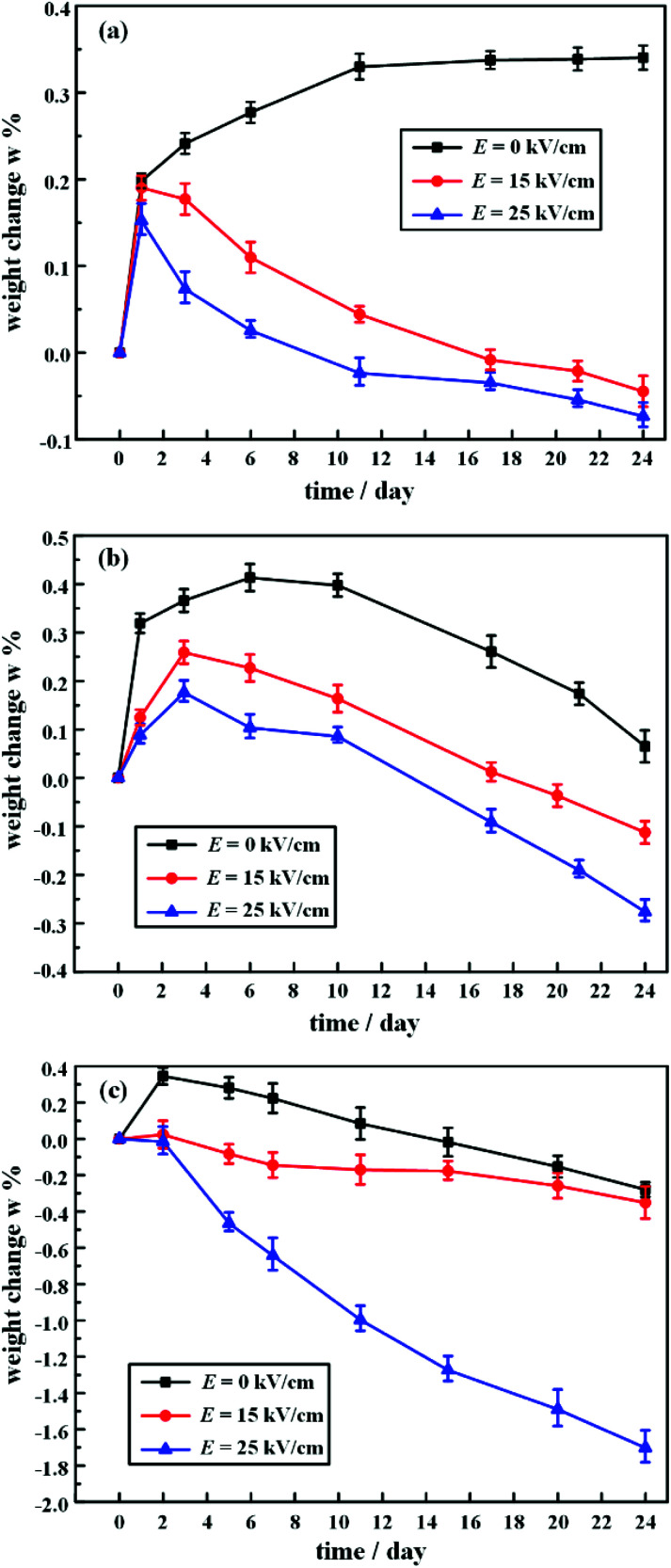
Relative weight change of HTV SR after electrical-hydrothermal aging under different electrical field intensity at (a) 30 °C, (b) 60 °C, and (c) 80 °C.

Fig. 4 shows the relative weight change of HTV SR after electrical-hydrothermal aging under different electric field intensity at varying temperatures. As shown in Fig. 4, in the absence of an electric field, the weights of HTV SR specimens immersed in saline solutions at 30 °C increased rapidly at first and then slowly increased within 24 days. However, when immersed in saline solutions at 60 °C and 80 °C, the weight of HTV SR increased rapidly and then decreased. This phenomenon might result from the increase of temperature and the invasion of water accelerating the hydrothermal aging effect of the HTV SR specimen. The relative weight change of HTV SR specimen depended on the competition between the invasion of water and the extraction of the low-molecular-weight (LMW) substances and the filler particles. Both process was influenced by water temperature. During the immersion for 24 days at 30 °C, the weight increased because of the invasion of water over counteracted the weight decrease caused by the extraction of the LMW substances and the filler particles. However, at 60 °C and 80 °C, the weight of HTV SR decreased rapidly because the oxidation decomposition of HTV SR specimen and the extraction of the LMW substances and the filler particles over counteracted the weight increase due to the diffusion of water into HTV SR.

When considering the electric field, the weight of the HTV SR samples was obviously decreased. With the increase of electric field intensity, the final mass loss rate was significantly higher than that of the HTV SR samples without an electric field. The final relative weight change of the hydrothermal aging samples at 80 °C was 0.27%, while the relative weight change rate reached −1.71% under the 80 °C and 25 kV cm^−1^ electrical-hydrothermal aging condition. Therefore, there was a synergistic effect of mutual enhancement of electrical-hydrothermal aging process, which significantly accelerated the weight reduction of HTV SR specimens. The presence of an electric field accelerated the hydrothermal aging process, which in turn accelerated the deterioration of the electric field to HTV SR samples.

### 3.2 AC breakdown studies

The Weibull distribution, proposed by the Swedish physicist Weibull in 1939, has been widely used to represent breakdown voltages.^18–20^ It reflected the failure probability of a dielectric being broken down at a certain electric field strength *E*. In this test, the two-parameter Weibull distribution was applied to evaluate the statistical law of the breakdown behavior of HTV SR under alternating electric fields. The function can be expressed as:^15^

2 *P*(*E*) = 1 − exp(−(*E*/*E*_0_)^*β*^)

where *P*(*E*) was the cumulative failure probability of breakdown; *E* was the breakdown field strength of HTV SR; *β* was the shape parameter related to the shape or the width of the distribution; *E*_0_ represented the breakdown field strength at *P*(*E*) = 63.2%. To get *E*_0_, the eqn (2) can be written as follows:

3 log_10_(−ln(1 − *P*(*E*))) = *β* log_10_ *E* − *β* log_10_ *E*_0_

To find the value of *P*(*E*), the Bernard estimator was given by ref. 21,4
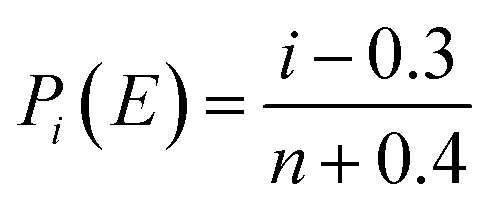
where *i* was the number of measurements in the ascending order of *E*; *n* was the total number of breakdown field strength tests per sample, and each HTV SR specimen was tested 5 times in this experiment.

**Fig. 5:**
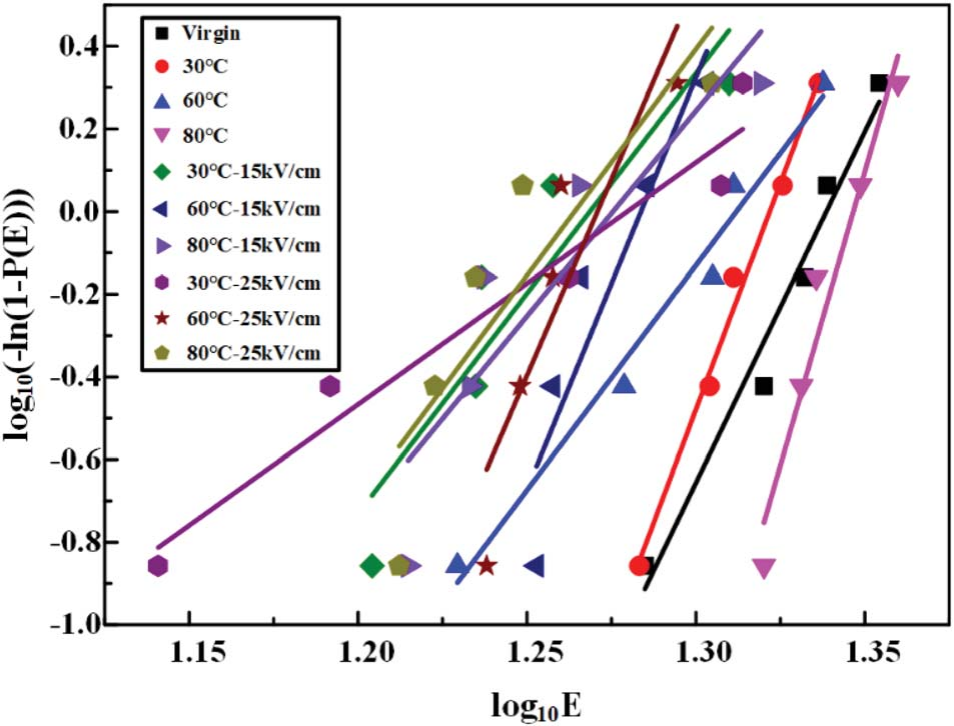
Curve of the Weibull statistical distribution of the breakdown field strength of before and after aging samples.

**Fig. 6:**
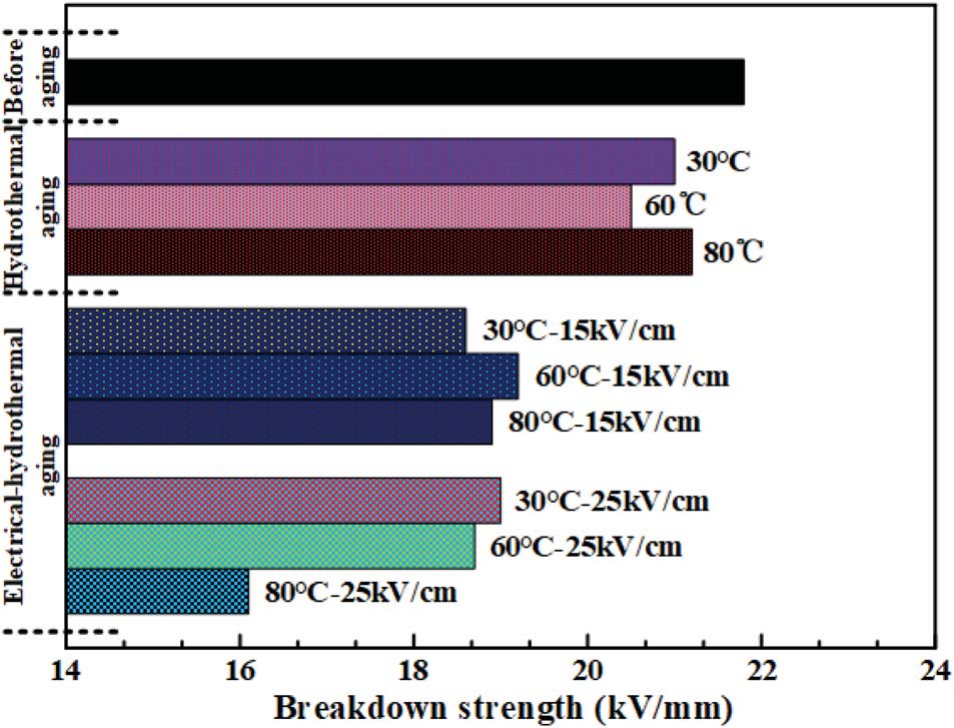
The breakdown field strength of HTV SR specimens under different aging conditions.

The AC breakdown characteristic of all samples under Weibull distribution is presented in Fig. 5. According to eqn (3), the value of the shape parameter *β* was calculated and listed in Table 2. Coppard^22^*et al.* believed that the shape parameter *β* represented the distribution of defects and filling particles. However, as seen in Table 2, the value of *β* did not show obvious regularity but presented a great fluctuation. It indicated that there were complex reactions in HTV SR after hydrothermal and electrical-hydrothermal aging processes. Due to high temperature and high electric field, severe degradation occurred in HTV SR and various physical and chemical defects were produced. Therefore, the change in the shape parameter *β* showed large variation in dispersity of electrical weak points in the aging samples. Then the value of breakdown field strength under different aging conditions is shown in Fig. 6. As shown in Fig. 6, compared to the virgin sample and hydrothermal aging sample, the breakdown field strength of the electrical-hydrothermal (80 °C – 25 kV cm^−1^) sample was significantly decreased from 21.8 kV mm^−1^ to 16.1 kV mm^−1^. It indicated that there was obvious synergistic aging effect of electric, water and temperature. The change of breakdown field strength caused by electrical-hydrothermal aging was much greater than that caused by hydrothermal aging. The combined effect significantly accelerated the decrease of breakdown field strength. To further explore the combined aging effect, the microscopic analysis was studied below.

**Table 2:** Shape parameter *β* of unaged and aged HTV SR specimens

Sample	Virgin	Hydrothermal aging (°C)			Electrical-hydrothermal aging (°C – kV cm^−1^)					
		30	60	80	30–15	60–15	80–15	30–25	60–25	80–25
Shape parameter *β*	17.0	21.9	10.9	28.5	10.6	19.9	9.9	5.9	19.0	10.9

### 3.3 Physicochemical properties and mechanical properties analysis

#### 3.3.1 SEM and EDAX analysis

**Fig. 7:**
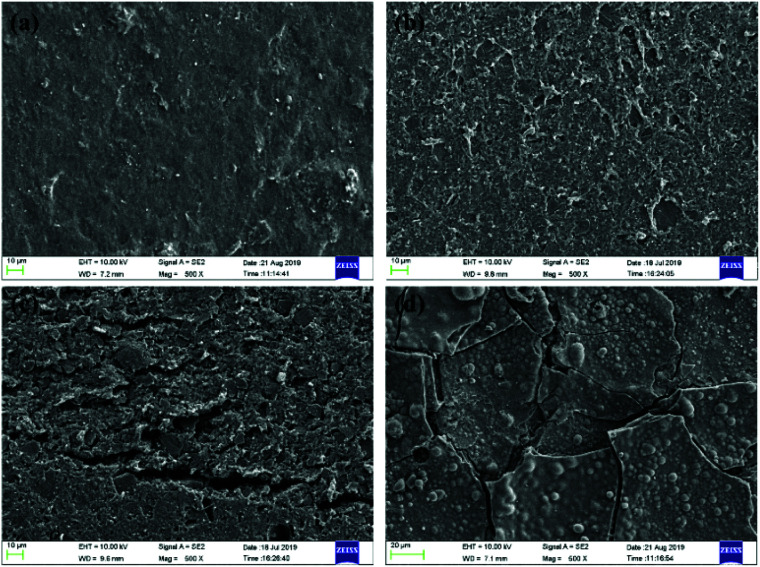
SEM photos of HTV SR specimens before and after electrical-hydrothermal aging in varying electrical field intensity at 80 °C for 24 days: (a) virgin HTV SR, (b) 0 kV cm^−1^, (c) 15 kV cm^−1^, (d) 25 kV cm^−1^.

**Fig. 8:**
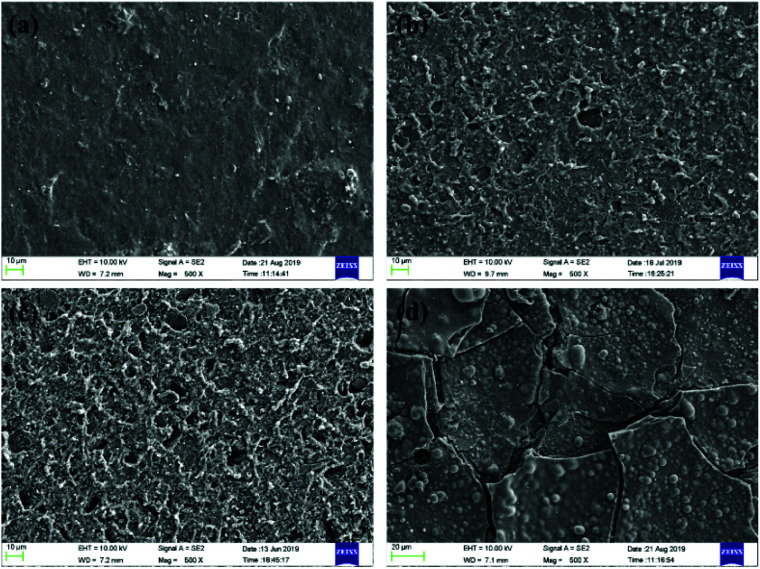
SEM photos of HTV SR specimens after electrical-hydrothermal aging at varying temperature under 25 kV cm^−1^ for 24 days: (a) virgin HTV SR, (b) 30 °C, (c) 60 °C, (d) 80 °C.

Fig. 7 and 8 present the SEM photos of HTV SR specimens before and after electrical-hydrothermal aging at varying electrical field intensity and varying temperatures for 24 days. It showed that the surface of the virgin HTV SR was smooth, on which there were no holes, cracks and obvious particles. Under the hydrothermal aging condition of 80 °C, the surface of HTV SR samples became rough and appeared a few holes. However, cracks appeared on HTV SR with the addition of electric field. When the electric field strength reached 25 kV cm^−1^, derived cracks developed on the surface of HTV SR in different orientation. It reflected that water tended to diffuse into the HTV SR at 80 °C, making it easier for electrons to inject into the material and accelerating the chain scission and the degradation process. Due to the irregularity of the self-diffusion of water, cracks appeared and developed in different directions after electrical-hydrothermal aging. Furthermore, the phenomenon was more obvious with the increase of electric field strength. Similarly, the rise of temperature promoted the growth of cracks and the increase of holes under the condition of electrical-hydrothermal aging at 25 kV cm^−1^. It was due to the synergism of electrical-hydrothermal aging, which accelerated the decomposition of PDMS molecules and the dissolution of fillers, resulting in severe degradation of HTV SR specimen.

**Fig. 9:**
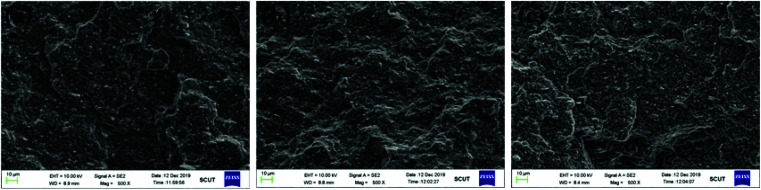
SEM photos of the cross-sections of HTV SR specimens before and after aging in different conditions for 24 days: (a) virgin HTV SR, (b) hydrothermal aging at 80 °C, (c) electrical-hydrothermal aging at 80 °C – 25 kV cm^−1^.

To examine the cross-sections of the HTV SR, SEM photos of specimens aging in different conditions to verify the depth of the damage was presented in Fig. 9. It indicated that the cross-sections of the virgin HTV SR was relatively smooth and presented little cracks, holes and particles compared to the aging samples. Then the cross-section of the HTV SR specimen became rough after hydrothermal aging. With the addition of electric field, a large number of microscopic cracks and holes appeared in the HTV SR specimen in Fig. 9(c). It can be concluded that the electrical-hydrothermal aging degradation occurred not only on the surface but also in the interior. However, the degradation on the surface showed much more serious than the interior. It indicated that the initial deterioration process began on the surface of the samples.

**Fig. 10:**
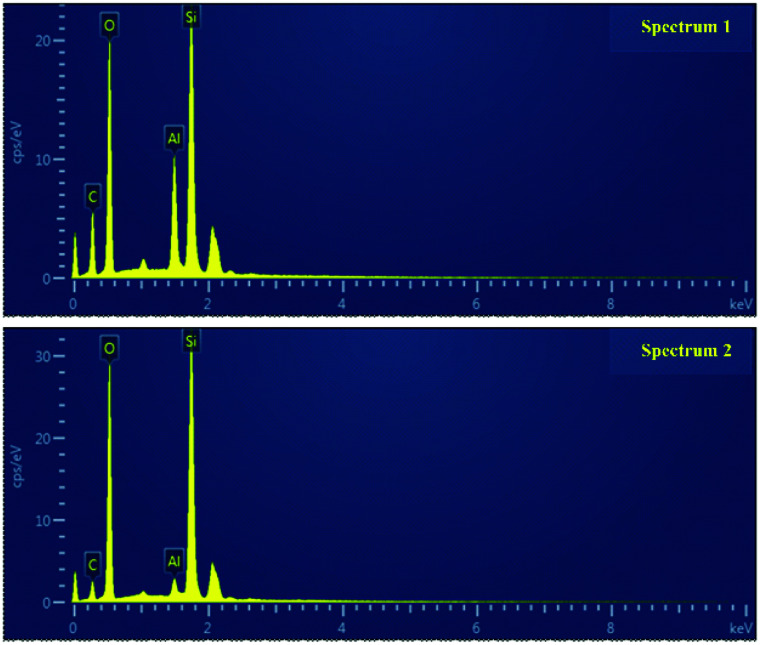
EDAX of the virgin HTV SR (spectrum 1) and the electrical-hydrothermal aged (80 °C – 25 kV cm^−1^) HTV SR (spectrum 2).

At the same time, EDAX was conducted to recognize the elements present over the HTV SR surface. Fig. 10 presents the EDAX of the virgin HTV SR (spectrum 1) and the electrical-hydrothermal aged (80 °C – 25 kV cm^−1^) HTV SR (spectrum 2). Because the test mainly studied the variation rules of surface elements on HTV SR after electrical-hydrothermal aging, various trace elements in the filler were neglected. As shown in Fig. 10, silicon (Si), carbon (C), oxygen (O) and aluminum (Al) were the main elements on the surface of the HTV SR specimen. After electrical-hydrothermal aging (80 °C – 25 kV cm^−1^) for 24 days, the content of elements C and Al decreased significantly. It represented the side chain scission of PDMS molecules, and the LMW substances would be extracted, resulting in a decline of C content. Moreover, Al content was also significantly reduced due to acidic substances generated by discharge. The generated acidic substances would react with the flame retardant aluminum hydroxide. The analysis could be further proved in the FTIR below.

#### 3.3.2 FTIR analysis

**Fig. 11:**
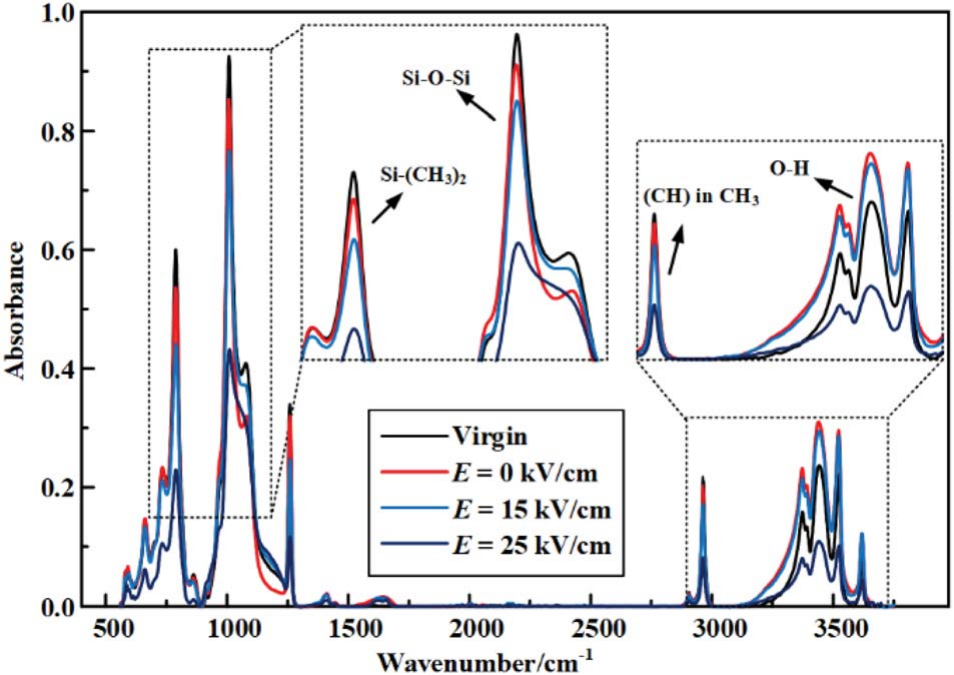
FTIR of HTV SR before and after electrical-hydrothermal aging at 80 °C for 24 days (varying electric field intensity).

**Fig. 12:**
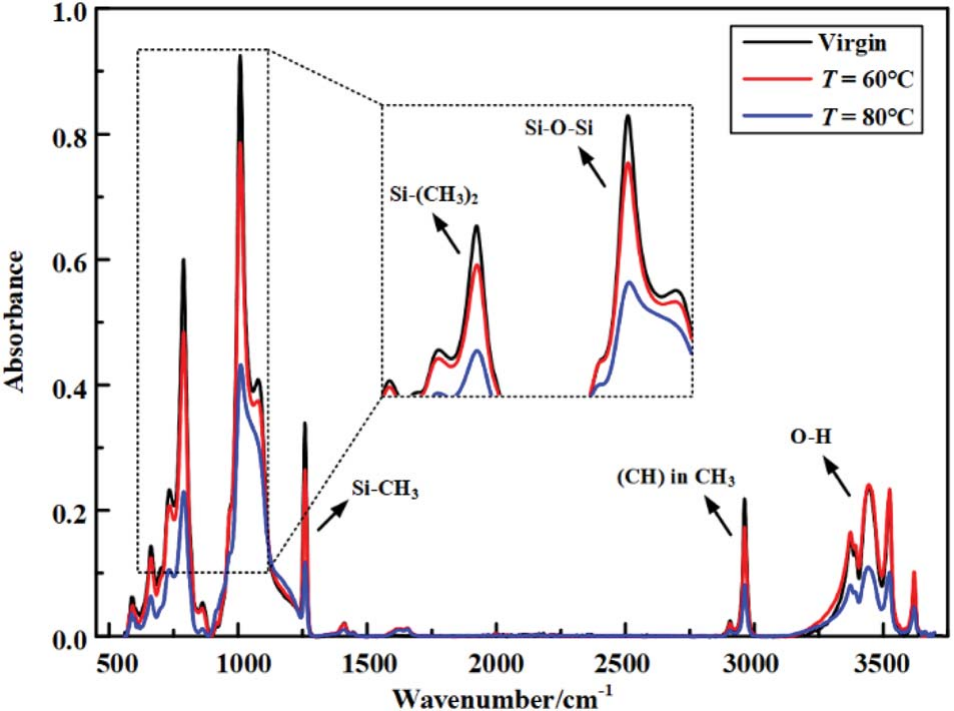
FTIR of HTV SR before and after electrical-hydrothermal aging at 25 kV cm^−1^ for 24 days (varying temperature).

After hydrothermal aging and electrical-hydrothermal aging for 24 days, the IR absorbance spectra for the fresh and after aged specimens were obtained (Fig. 11 and 12). Table 3 lists the characteristic IR absorption bands in HTV. Fig. 11 shows the FTIR of HTV SR before and after electrical-hydrothermal aging at 80 °C for 24 days. As shown in Fig. 11, with the increase of electric field intensity, the peak of the main chain Si–O–Si, side chain Si–CH_3_ and Si–(CH_3_)_2_ of PDMS in HTV SR specimen showed a downward trend. When the electric field intensity reached 25 kV cm^−1^, the absorption peak of the main chain and each side chain showed a remarkable reduction. For the hydroxyl absorption peak in the wavenumber range from 3200 cm^−1^ to 3700 cm^−1^, it fluctuated with the increase of field intensity. It was caused by the complex reaction processes such as oxidation,^14^ cracking,^23^ crosslinking^24,25^ and condensation^26^ that occurred in the electrical-hydrothermal aging process of HTV SR samples, resulting in the fluctuating tendency of hydroxyl peak. Fig. 12 shows the FTIR of HTV SR before and after electrical-hydrothermal aging at 25 kV cm^−1^ for 24 days. Similar to the downward trend in Fig. 11, when the electric field intensity was 25 kV cm^−1^, the peak of each organic group decreased significantly with the increase of the temperature in electrical-hydrothermal aging processes. Therefore, it indicated that the PDMS on the HTV SR surface severely degraded when the electric field intensity or the temperature increased.

**Table 3:** Characteristic IR absorption bands in HTV

Bond	Wave number/cm^−1^
O–H	3700–3200
(C–H) in CH_3_	2960
Si–CH_3_	1270–1255
Si–O–Si	1100–1000
Si–(CH_3_)_2_	840–790

#### 3.3.3 Tensile properties analysis

**Fig. 13:**
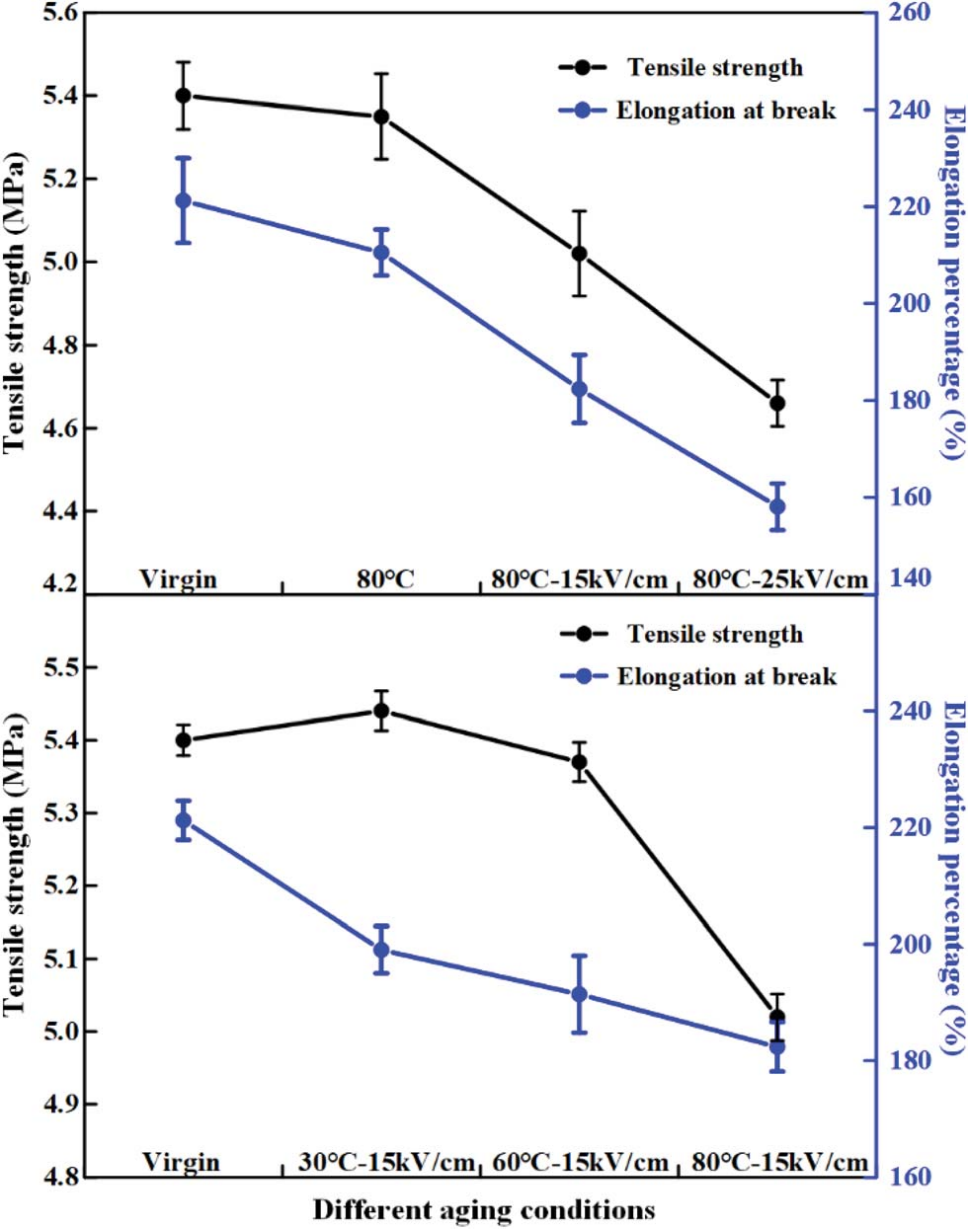
Tensile properties of HTV SR before and after aging in different conditions.

**Fig. 14:**
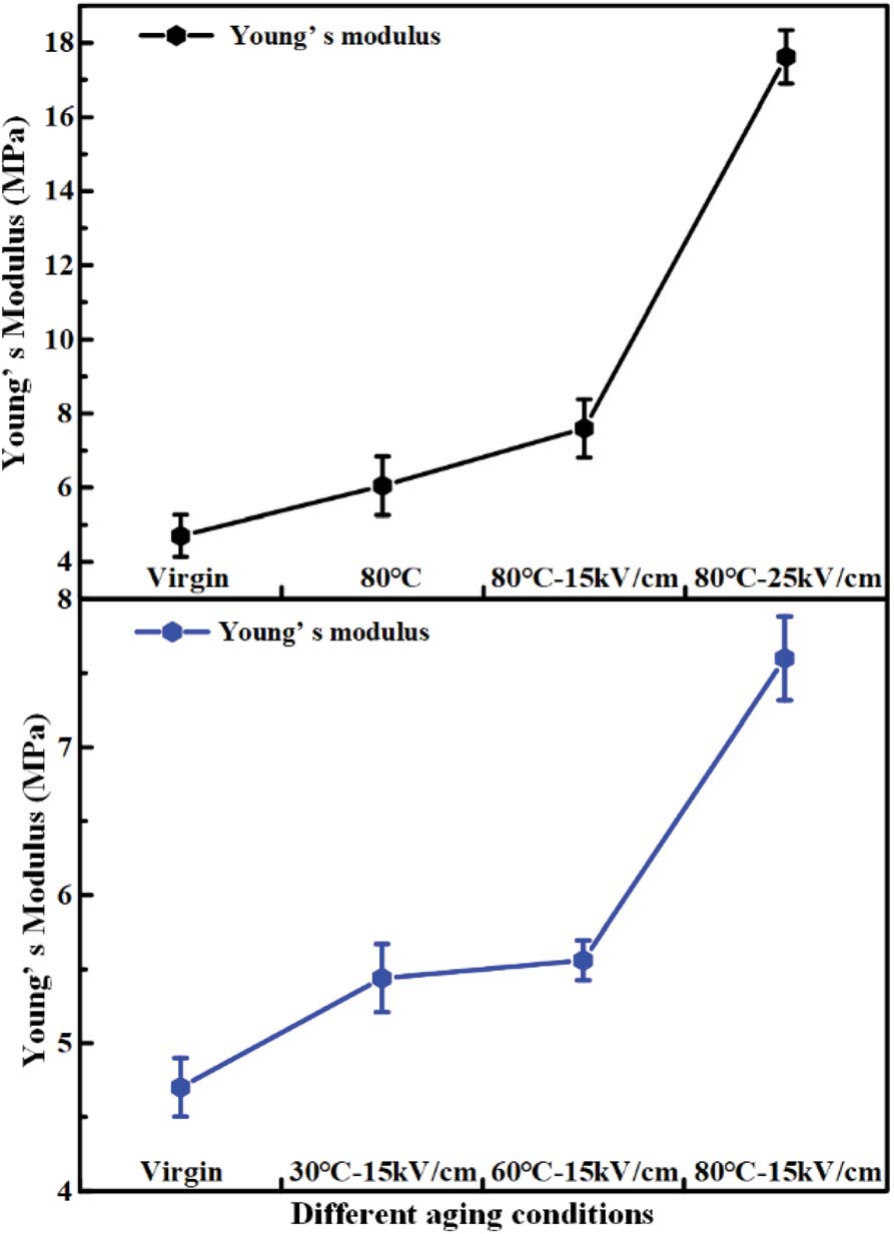
Young's modulus of HTV SR before and after aging in different conditions.

The mechanical property is an important index to evaluate the aging characteristics of insulating materials. Fig. 13 and 14 present the change of the tensile strength, the elongation at break and the Young's modulus in different aging conditions after hydrothermal and electrical-hydrothermal aging. As shown in Fig. 13 and 14, the tensile strength and the elongation at break decreased and the Young's modulus increased with the increase of the electric field strength. In the presence of an electric field, the change of the tensile strength, the elongation at break and the Young's modulus of the aging HTV SR specimens were much greater than that of the hydrothermal aging samples. The deterioration in tensile properties after aging is normally related to linkages scission and main chain scission. According to FTIR results, the peak of the main chain Si–O–Si, side chain Si–CH_3_ and Si–(CH_3_)_2_ of PDMS in HTV SR specimen showed a downward trend. The crosslinking structure was destroyed after electrical-hydrothermal aging. However, the change of tensile strength and Young's modulus at varying temperature presented different trends in electrical-hydrothermal aged specimens. At 30 °C – 15 kV cm^−1^ and 60 °C – 15 kV cm^−1^, the tensile strength and the Young's modulus did not show obvious change. When temperature reached 80 °C, the tensile strength significantly decreased and the Young's modulus significantly increased. It revealed that when the temperature did not reach the threshold, the temperature would not promote the electrical aging process.

### 3.4 Frequency response analysis

**Fig. 15:**
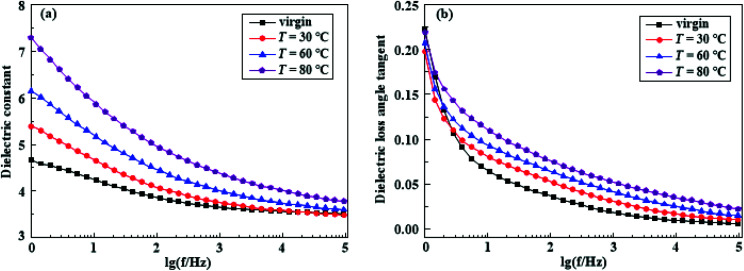
The dielectric constant (a) and the dielectric loss angle tangent (b) of HTV SR before and after electrical-hydrothermal aging at 15 kV cm^−1^ for 24 days (varying temperature).

In this section, the data of 80 °C and 15 kV cm^−1^ after electrical-hydrothermal aging for 24 days were selected for analysis. Fig. 15(a) and (b) shows the change of dielectric constant and the dielectric loss of HTV SR at varying temperature after electrical-hydrothermal aging at 15 kV cm^−1^ for 24 days.

As shown in Fig. 15(a), it indicated that the dielectric constant of the aging samples in the whole frequency range significantly increased with the rise of temperature, especially at low frequency. However, in the high frequency band (10^4^ Hz to 10^5^ Hz), the curve tended to a constant, which might be caused by electron polarization or molecular chain vibration of HTV SR media under the influence of the electric field. Fig. 15(b) shows that the dielectric loss of HTV SR specimen increased with the rise of temperature in the middle and high frequency band, while in the low frequency band, the dielectric loss of the electrical-hydrothermal aging samples was lower than that of the initial samples.

This phenomenon can be attributed to the following aspects. First of all, according to Debye relaxation theory, the dielectric loss of HTV SR samples at low frequency band is mainly caused by the DC conductance loss. With the increase of frequency, the polarization loss of different relaxations tends to play an important role and gradually becomes dominant. Secondly, in literature,^27^ the dielectric properties of silicone rubber sleeve were measured by the non-contact dielectric spectrum, and the various relaxation processes of silicone rubber were analyzed in detail by using the modified Cole–Cole model. The polarization processes were divided into macroscopic interface polarization, microscopic interface polarization and dipole polarization. Considering that the contact dielectric spectrum was selected in this test, the macroscopic interface polarization process can be neglected. Therefore, the dielectric loss of silicone rubber samples was mainly caused by the microscopic interface polarization and dipole polarization. According to the SEM analysis, the polarization loss at the microscopic interface enlarged with the increase of physical and chemical defects such as holes and cracks on the surface of HTV SR and the loss of packing. Combined with the infrared spectrum characteristics, it can be seen that the rise of temperature aggravated the damage degree of the molecular main chain and decomposed to produce more LMW substances, leading to the increase of dipoles and dipole polarization loss.

**Fig. 16:**
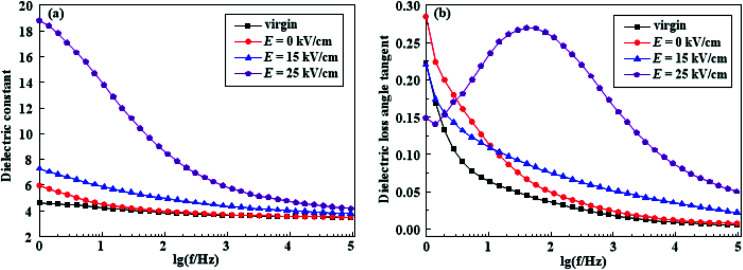
The dielectric constant (a) and the dielectric loss angle tangent (b) of HTV SR before and after electrical-hydrothermal aging at 80 °C for 24 days (varying electric field intensity).

Fig. 16(a) and (b) shows the change of dielectric constant and the dielectric loss of HTV SR under different electric field intensity after electrical-hydrothermal aging at 80 °C for 24 days. As seen in Fig. 16(a), the dielectric constant behave similarly for the HTV SR of the aged and the virgin sample, and increased with decreasing frequency. Especially in the low frequency band, the increase became faster because of the space charge polarization or the effect of interfacial polarization. Due to the appearance of various physical and chemical defects on the surface and inside of HTV SR, as well as the increase of particles shown by SEM results, the interface polarization was significantly enhanced. The highest observed values (dielectric constant) were for the samples after electrical-hydrothermal aging at 80 °C – 25 kV cm^−1^. As compared to the virgin sample, there was an obvious increase in dielectric constant almost along the entire frequency range for hydrothermal and electrical-hydrothermal aged HTV SR specimen. This might be due to the increase in mobility of the polymer chains by the breakage of bonds caused by applied electric and hydrothermal stress.

As regards the dielectric loss angle tangent, in Fig. 16(b), it presented different and interesting phenomenon. In the middle frequency region (10 Hz to 10^3^ Hz), there was an obvious dielectric loss peak in the HTV SR specimen after electrical-hydrothermal aging at 80 °C – 25 kV cm^−1^ for 24 days. It might be caused by the orientation of greater dipolar groups. A large amount of broken PDMS molecular chains led to the increase of free radicals in the material and the enhancement of dipole polarization, presenting the apparent loss peak in the dielectric frequency response. However, in the low frequency region, the dielectric loss angle tangent of the electrical-hydrothermal aging samples showed different change rules. Because there was another dielectric relaxation process in the dielectric loss curve at low frequency band in addition to the DC conductivity,^27^ and they worked together in the low frequency region of the dielectric loss curve.

### 3.5 Trap properties analysis

**Fig. 17:**
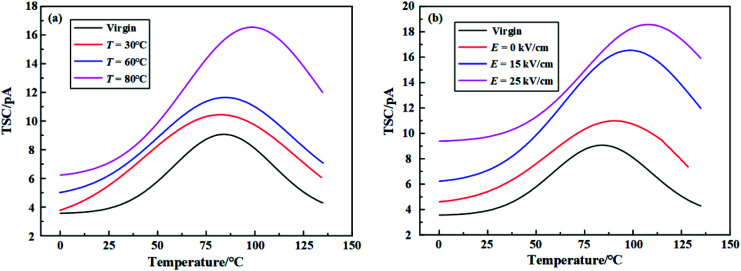
TSC measurement before and after electrical-hydrothermal aging for 24 days: (a) *E* = 15 kV cm^−1^, varying temperature; (b) *T* = 80 °C, varying electric field intensity.

It is commonly acknowledged that the aging performance of the polymer material is related to the accumulation of space charge or the trap characteristics. Fig. 17 shows the TSC curve of unaged, the hydrothermal aged and electrical-hydrothermal aged samples. In order to analyze trap properties, the amount of trap charge can be calculated by eqn (5). Then the amount of trap charge, the peak current and the peak current temperature can be listed in Table 3.5
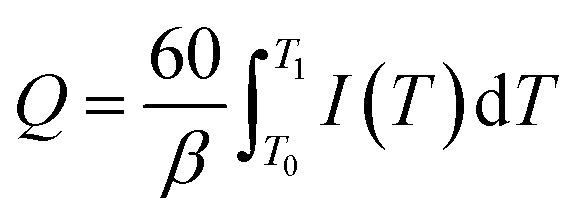


As shown in Fig. 17, it indicated that the TSC curve of all HTV SR samples presented a main current peak. With the increase of the temperature or the electric field strength of electrical-hydrothermal aging, the peak current increased continuously and tended to drift towards the high temperature region. As listed in Table 4, the trap charge of the electrical-hydrothermal aging samples was significantly more than that of the unaged and after hydrothermal aged samples. Similarly, the trap charge also increased with the rising temperature and electric field strength. Because there were more physical and chemical defects generated in the HTV SR specimen, and the deterioration degree was deepened, which led to the increase of trap and trap charge.

**Table 4:** TSC current peaks and trap parameters of unaged and aged HTV SR specimens

Sample	Virgin	Hydrothermal aging (°C)			Electrical-hydrothermal aging (°C – kV cm^−1^)					
		30	60	80	30–15	60–15	80–15	30–25	60–25	80–25
*Q* (nC)	9.60	9.96	10.80	11.17	12.24	13.80	18.24	15.00	16.56	21.36
Peak current (pA)	9.07	9.15	9.36	10.98	10.44	11.64	16.50	12.55	12.69	18.57
Peak temperature (°C)	84.01	78.97	75.49	90.35	82.35	84.84	98.49	94.45	97.38	107.73

However, it is almost difficult to obtain the trap density and trap energy level distribution of the HTV SR specimen directly before and after aging from Fig. 17. According to the TSC theory, the TSC caused by the de-trapping process of the captured charges can be expressed as the following function:^28^6
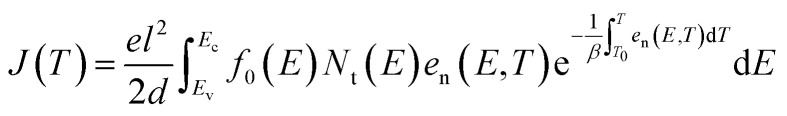
where *N*_t_ (*E*) is the distribution function, which is spatially uniform deep into the sample with a distance *l*. *e* is electronic charge quantity, *f*_0_ is the initial occupancy of a trap level and is a constant, *E* is the trap energy (trap depth). *e*_n_(*E*,*T*) = *v* exp(−*E*_t_/*kT*) is the emission rate of electrons at trap level *E* and temperature *T*. *k* is the Boltzmann constant, *d* is the sample thickness and *β* is the heating rate. *v* is commonly called the frequency factor or attempt-to-escape frequency, typically 10^12^ s^−1^ as suggested by Mott.^29^*E*_v_ and *E*_c_ are the valence band top energy and conduction band bottom energy, respectively.

**Fig. 18:**
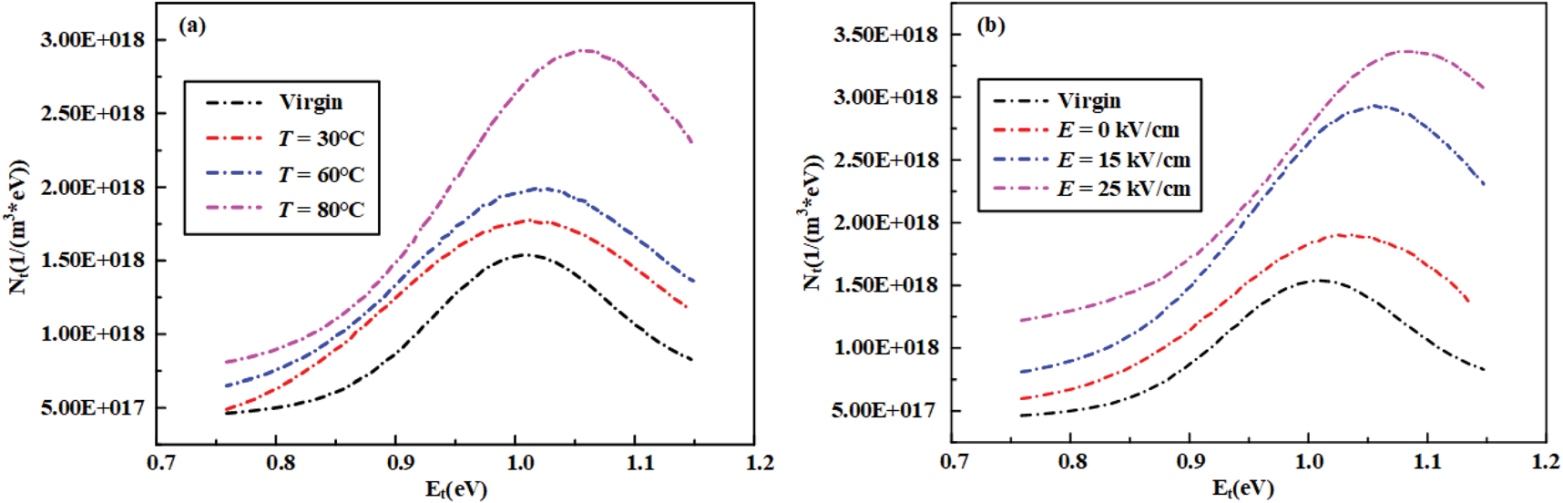
Trap level distributions before and after electrical-hydrothermal aging for 24 days: (a) *E* = 15 kV cm^−1^, varying temperature; (b) *T* = 80 °C, varying electric field intensity.

Then, the trap level distribution from TSC measurement can be calculated by eqn (6) based on MATLAB numerical calculation. Fig. 18 shows the trap level distributions of the virgin and aging samples. It shows that the trap density of the virgin was relatively low. With the rise of temperature and electric field intensity, the trap density increased and the trap energy level corresponding to the peak of trap density became deeper. The peak density of traps could reach about 2.5 times larger than that of the virgin sample under the condition of 80 °C – 25 kV cm^−1^ electrical-hydrothermal aging. The phenomenon can be explained by the following aspects. Firstly, with the combination of electrical-hydrothermal aging, more defects were produced inside the HTV SR, which enhanced the electron trapping ability and increased the trap density. Secondly, it is well known that traps can be divided into deep traps and shallow traps according to the trap level. The current peak at low temperature is mainly contributed by the charge decapitation in shallow traps, while the current peak at high temperature corresponds to the charge decapitation in deep traps. As shown in Fig. 18, compared to the virgin sample, more deep traps were generated in the process of electrical-hydrothermal aging. Deep traps bound carrier migration, leading to local charge accumulation and rising local electric field strength. When the threshold was exceeded, the polymer breakdown might occur finally.

### 3.6 Discussion

According to the trap theory of solid dielectric, the energy band of insulators can be divided into the conduction band, forbidden band and valence band. The charges injected by the electrode can pass through the potential barrier into the conduction band through the Schottky effect and be captured by the trap in the forbidden band.^30^ The relaxation time of captured charges in traps is mainly determined by trap energy level and trap energy density. The larger trap energy level and density, the longer the relaxation time would be. Electrons captured by traps must overcome the larger trap barrier to be released. Due to the increase of trap energy level and trap density after electrical-hydrothermal aging, it is easier to form charge accumulation. When charges accumulate to a certain critical value, the breakdown happens.

**Fig. 19:**
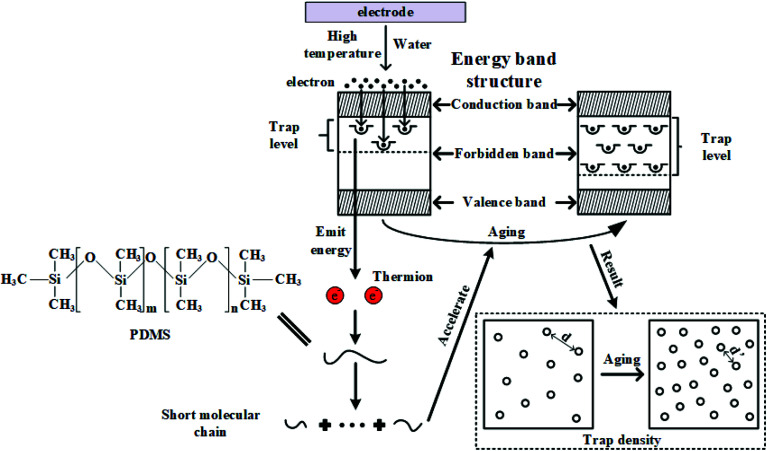
Electrical-hydrothermal combined aging mechanism.

Combined with the test results above, the mechanism of electrical-hydrothermal aging can be summarized in Fig. 19. First of all, when the HTV SR specimen is subjected to hydrothermal aging effects, the main and side chains scission on PDMS molecular chain occurred, producing free radical and LMW substances. With the aging process going on, more physical and chemical defects are formed in the material. The oxidation decomposition reaction in the aging process promotes the formation of more free molecules and traps, leading to the increase of dielectric constant, loss tangent tan *δ*, trap energy level and trap density.

Then, when the electric field is added, electrons would be injected into the HTV SR from the electrode, and some electrons are trapped by the polymer. The trapped electron becomes the recombination center of the hole, releasing large amounts of energy and transferring it to other electrons to produce thermions. The hydrothermal aging condition provides convenience for electron injection. More thermions will bombard the molecular chain, creating more traps. On the other hand, the dipole polarization loss generates thermal power, which aggravates the deterioration of insulating materials. Therefore, according to the trap properties during the aging process, producing nanocomposites can be an available way. The addition of nanoparticles can reduce the number of traps in the material and enhance breakdown strength. By adjusting the charge transport characteristics, the breakdown probability can be lowered down.

## 4. Conclusions

This study focuses on the electrical-hydrothermal aging experiment of HTV SR based on the high temperature and high humidity environment all over the world. Some important conclusions can be summarized as follows:

(1) Compared with hydrothermal aging samples, the relative weight, tensile strength, elongation at break and breakdown field strength significantly decreased after electrical-hydrothermal aging. The surface and interior of HTV SR were severely degraded, which was reflected in the scission of a large number of PDMS molecular chains, and the loss of flame retardant Al(OH)_3_.

(2) The dielectric constant and dielectric loss of HTV SR samples significantly increased with the rise of the aging temperature and electric field strength. When aged at 80 °C and 25 kV cm^−1^, there was an obvious dipole polarization loss peak that occurred due to the breaking of molecular chains and the increase of dipoles.

(3) The electrical-hydrothermal aging has a synergistic effect. The presence of temperature and water facilitates the electron injection and deepens the trap energy level and trap density of HTV SR, which in turn increases the probability of electron entrapment, accelerating the deterioration process of HTV SR.

## Conflicts of interest

There are no conflicts to declare.

## Supplementary Material

## References

[cit1] Bleszynski M., Kumosa M. (2017). Silicone rubber aging in electrolyzed aqueous salt environments. Polym. Degrad. Stab..

[cit2] Bretuj W., Fleszynski J., Wieczorek K., Ziaja J. (2010). Influence of composite insulators sheds diameter on ageing resistance in laboratory research. Przegl. Elektrotech..

[cit3] Solis-Ramos E., Kumosa M. (2017). Synergistic effects in stress corrosion cracking of glass reinforced polymer composites. Polym. Degrad. Stab..

[cit4] Yang S. F., Jia Z. D., Ouyang X. G., Wang S. H. (2018). Inhibition of algae growth on HVDC polymeric insulators using antibiotic-loaded silica aerogel nanocomposites. Polym. Degrad. Stab..

[cit5] Wang J. J., Lee W. H., Ho J. C., Hu T. S. (2009). High performance OTFTs using surface-modified nanocomposite dielectric gate insulator. J. Mater. Sci.: Mater. Electron..

[cit6] Wang R., Xie C. Z., Zeng L. L., Xu H. S. (2019). Thermal decomposition behavior and kinetics of nanocomposites at low-modified ZnO content. RSC Adv..

[cit7] Yang Q., Sima W. X., Deng J. Z., Sun C. X., Hu J. L. (2012). Shed Configuration Optimization for Ice-Covered Extra High Voltage Composite Insulators. J. Adhes. Sci. Technol..

[cit8] Yuan C., Xie C. Z., Li L. C., Xu X. D., Gubanski S. M. (2017). Surface Potential Decay on Material Samples Taken from In-service Aged HVDC Silicone Rubber Composite. IEEE Trans. Dielectr. Electr. Insul..

[cit9] Yuan C., Xie C. Z., Li L. C., Xu X. D., Gubanski S. M., Zhou Y., Li Q., He J. L. (2019). Space Charge Behavior in Silicone Rubber from In-Service Aged HVDC Composite Insulators. IEEE Trans. Dielectr. Electr. Insul..

[cit10] Zhu Y., Otsubo M., Honda C., Tanaka S. (2006). Loss and recovery in hydrophobicity of silicone rubber exposed to corona discharge. Polym. Degrad. Stab..

[cit11] Song W., Shen W. W., Zhang G. J., Song B. P., Lang Y., Su G. Q., Mu H. B., Deng J. B. (2015). Aging Characterization of High Temperature Vulcanized Silicone Rubber Housing Material Used for Outdoor Insulation. IEEE Trans. Dielectr. Electr. Insul..

[cit12] Verma A. R., Reddy B. S., Chakraborty R. (2018). Multistress Aging Studies on Polymeric Insulators. IEEE Trans. Dielectr. Electr. Insul..

[cit13] Montanari G. C., Mazzanti G., Simoni L. (2002). Progress in electrothermal life modeling of electrical insulation during the last decades. IEEE Trans. Dielectr. Electr. Insul..

[cit14] Chen C., Jia Z. D., Ye W. A., Guan Z. C., Li Y. Z. (2017). Thermo-oxidative Aging Analysis of HTV Silicone Rubber Used for Outdoor Insulation. IEEE Trans. Dielectr. Electr. Insul..

[cit15] Wang Z., Jia Z. D., Jiao J. K., Guan Z. C. (2016). Influence of Water, NaCl Solution, and HNO_3_ Solution on High-Temperature Vulcanized Silicone Rubber. IEEE Trans. Dielectr. Electr. Insul..

[cit16] Ali M., Hackam R. (2008). Effects of Saline Water and Temperature on Surface Properties of HTV Silicone Rubber. IEEE Trans. Dielectr. Electr. Insul..

[cit17] Zhu Y., Haji K., Otsubo M., Honda C. (2006). Surface degradation of silicone rubber exposed to corona discharge. IEEE Trans. Plasma Sci..

[cit18] Pelissou S. , Benca P. and Gross L. H., Electrical properties of metallocene polyethylene, Proceedings of the 2004 IEEE International Conference on Solid Dielectrics, vol. 1–2, 2004, pp. 466–469

[cit19] Kauerauf T., Degraeve R., Cartier E., Soens C., Groeseneken G. (2002). Low Weibull slope of breakdown distributions in high-k layers. IEEE Electron Device Lett..

[cit20] Montanari G. C., Mazzanti G., Cacciari M., Fothergill J. C. (1997). In search of convenient techniques for reducing bias in the estimation of Weibull parameters for uncensored tests. IEEE Trans. Dielectr. Electr. Insul..

[cit21] Montanari G. C., Mazzanti G., Cacciari M., Fothergill J. C. (1997). In search of convenient techniques for reducing bias in the estimation of Weibull parameters for uncensored tests. IEEE Trans. Dielectr. Electr. Insul..

[cit22] Coppard R. W., Bowman J., Dissado L. A. (1990). *et al.*, The effect of aluminium inclusions on the dielectric breakdown of polyethylene. J. Phys. D: Appl. Phys..

[cit23] Kim J., Chaudhury M. K., Owen M. J., Orbeck T. (2001). The mechanisms of hydrophobic recovery of polydimethylsiloxane elastomers exposed to partial electrical discharges. J. Colloid Interface Sci..

[cit24] Hollahan J. R., Carlson G. L. (1970). Hydroxylation of Polydimethylsiloxane Surfaces by Oxidizing Plasmas. J. Appl. Polym. Sci..

[cit25] Delman A. D., Landy M., Simms B. B. (1969). Photodecomposition of Polydimethylsiloxane. J. Polym. Sci., Part A-1: Polym. Chem..

[cit26] Chainet F., Meur L. L., Lienemann C. P. (2013). *et al.*, Characterization of silicon species issued from PDMS degradation under thermal cracking of hydrocarbons: part 1–gas samples analysis by gas chromatography-time of flight mass spectrometry. Fuel.

[cit27] Yuan C., Xie C. Z., Li L. C., Xu X. D., Gubanski S. M., He Z. L. (2016). Dielectric Response Characterization of In-service Aged Sheds of (U) HVDC Silicone Rubber Composite Insulators. IEEE Trans. Dielectr. Electr. Insul..

[cit28] Tian F. Q., Bu W. B., Shi L. S., Yang C., Wang Y., Lei Q. Q. (2011). Theory of modified thermally stimulated current and direct determination of trap level distribution. J. Electrost..

[cit29] Mott N. F. , and Gurney R. W., Electronic Processes in Ionic Crystals, Oxford University Press, London, 1948

[cit30] Wang R., Xie C. Z., Luo S. K., Gou B., Xu H. S., Zeng L. L. (2019). The influence mechanism of nanoparticles on the dielectric properties of epoxy resin. RSC Adv..

